# Supplementary material for: Transcriptional and Epigenetic Alterations in the Progression of Non-Alcoholic Fatty Liver Disease and Biomarkers Helping to Diagnose Non-Alcoholic Steatohepatitis
Source: Biomedicines. 2023 Mar 21;11(3):970. doi: 10.3390/biomedicines11030970 (PMC10046227; doi:10.3390/biomedicines11030970)
Supplement: Supplementary file 1 [file biomedicines-11-00970-s001.zip › Approval of Animal Ethical and Welfare.pdf]

动物实验伦理审查同意书

Approval of Animal Ethical and Welfare

张禾

经四川大学华西医院实验动物伦理委员会初审和复审审查后，您申报的课题
探索非酒精性脂肪肝模型鼠肝脏组织特定基因的表达模式

符合国家实验动物福利伦理的相关规定，给予通过。

伦理备案号：20220301147

Dear ____________________

Animal Ethics Committee have a meeting as scheduled, the participators have evaluated the care and use of animals described in the protocol of ____,

and find the procedures described as appropriate and acceptable.

Approval No. ____________________

华西医院实验动物伦理委员会主席
Signature of Chairman [illegible]

时间
Date 2022.3.2

四川大学华西医院 实验动物伦理委员会
