# Supplementary material for: Transcriptional and Epigenetic Alterations in the Progression of Non-Alcoholic Fatty Liver Disease and Biomarkers Helping to Diagnose Non-Alcoholic Steatohepatitis
Source: Biomedicines. 2023 Mar 21;11(3):970. doi: 10.3390/biomedicines11030970 (PMC10046227; doi:10.3390/biomedicines11030970)
Supplement: Supplementary file 1 [file biomedicines-11-00970-s001.zip › Supplementary Figure S1-S3 20221031.pdf]

Supplementary

Figures S1-S3

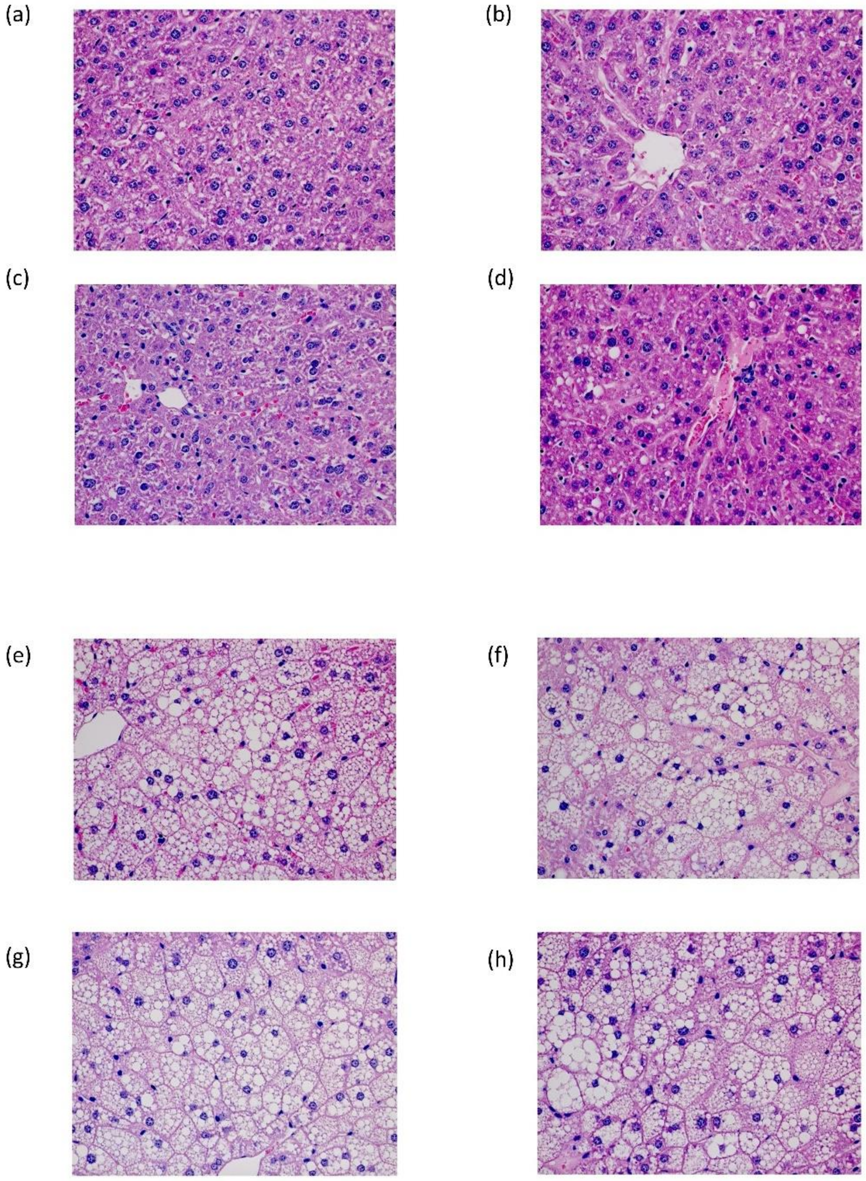

Figure S1. Liver histology of the mouse model. (**a**-**d**) Liver biopsy of NAFL sample 2-5 with hematoxylin-eosin staining, scale bar 20 μm ; (**e**-**h**) Liver biopsy of NASH sample 2-5 with hematoxylin-eosin staining, scale bar 20 μm.

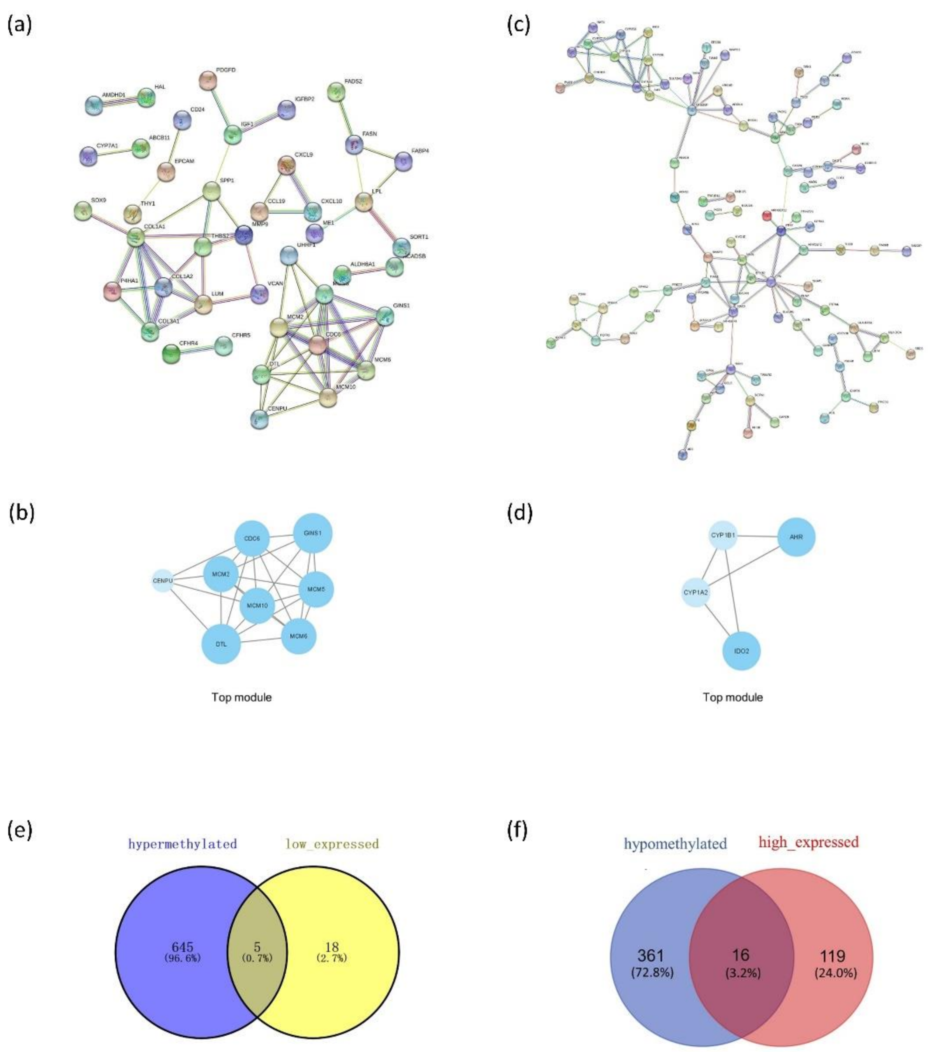

Figure S2. The network of PPI analysis. (**a**) PPI network of the DEGs in the Normal vs NASH group; (**b**) Top module of the DEGs in the Normal vs NASH group; (**c**) PPI network of the DMGs in the Normal vs NASH group; (**d**) Top module of the DMGs in the Normal vs NASH group; (**e**) Identification of hypermethylated and low expressed genes; (**f**) Identification of hypomethylated and high expressed genes.

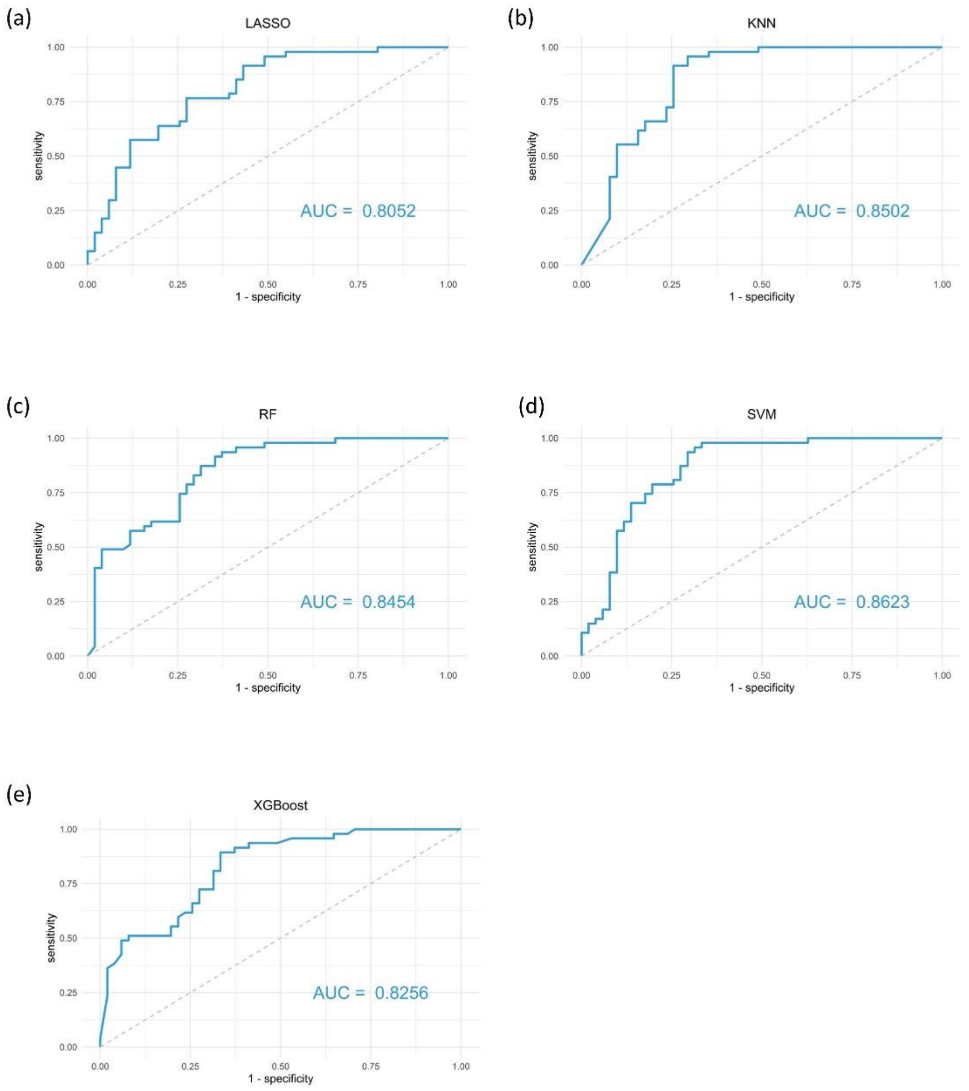

Figure S3. Receiver operating characteristic curves of different diagnostic models. (**a**) Receiver operating characteristic curve of the LASSO model; (**b**) Receiver operating characteristic curve of the KNN model; (**c**) Receiver operating characteristic curve of the RF model; (**d**) Receiver operating characteristic curve of the SVM model; (**e**) Receiver operating characteristic curve of the XGBoost model.
